# Genomic and epigenomic alterations in prostate cancer

**DOI:** 10.3389/fendo.2012.00128

**Published:** 2012-11-06

**Authors:** Anna M. Aschelter, Silvana Giacinti, Paola Caporello, Paolo Marchetti

**Affiliations:** Department of Oncology, Sant’Andrea Hospital, “Sapienza” University of RomeRome, Italy

**Keywords:** prostate cancer

## Abstract

Prostate cancer (PC) is the second most frequently diagnosed cancer and the second leading cause of cancer deaths in man. The treatment of localized PC includes surgery or radiation therapy. In case of relapse after a definitive treatment or in patients with locally advanced or metastatic disease, the standard treatment includes the androgen-deprivation therapy (ADT). By reducing the levels of testosterone and dihydrotestosterone under the castration threshold, the ADT acts on the androgen receptor (AR), even if indirectly. The effects of the ADT are usually temporary and nearly all patients, initially sensitive to the androgen ablation therapy, have a disease progression after an 18–24 months medium term. This is probably due to the selection of the cancer cell clones and to their acquisition of critical somatic genome and epigenomic changes. This review aims to provide an overview about the genetic and epigenetic alterations having a crucial role in the carcinogenesis and in the disease progression toward the castration resistant PC. We focused on the role of the AR, on its signaling cascade and on the clinical implications that the knowledge of these aspects would have on hormonal therapy, on its failure and its toxicity.

## INTRODUCTION

**FIGURE 1:**
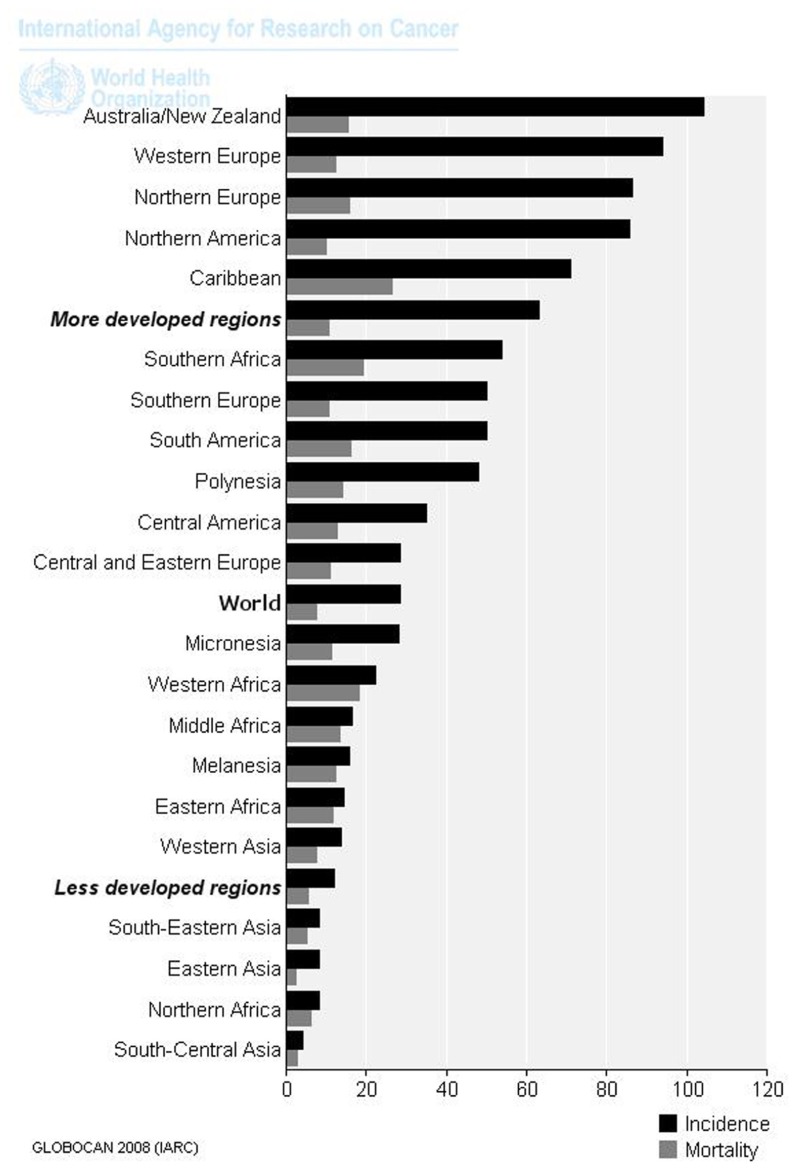
**Age-standardized rates (world) per 100,000 of PC in 2008 (data from Ferlay et al., 2010)**.

Prostate cancer (PC) is the second most frequently diagnosed cancer and the second leading cause (the first one is lung cancer) of cancer deaths in man. The estimate of new diagnoses and deaths from PC in 2012, in the United States, amounted to 241,740 and 28,170, respectively (American Cancer Society, 2012). In Italy, it has been estimated that every year 23,518 new PCs are diagnosed; deaths due to PC are 7,105 (AIRT, 1998–2002). PC incidence and mortality displays geographic variation, with high rates of incidence and mortality in the US and Western Europe, and low rates in Asia (Makridakis et al., 1997; **Figure 1**).

**FIGURE 2:**
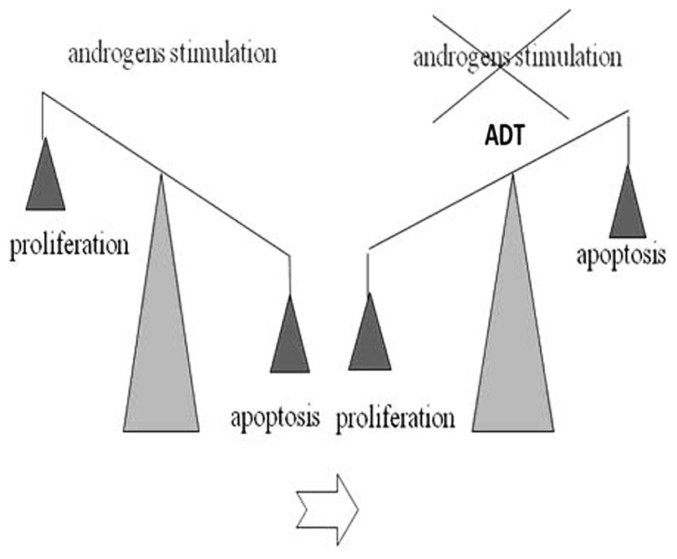
**Androgen-deprivation therapy (ADT) effect on proliferation/apoptosis PC cells: androgens stimulate the tumor cells proliferation and inhibit their apoptosis**. ADT reverses the equilibrium in favor of the apoptosis.

Data show that mortality from PC is relatively lower compared to the total number of yearly diagnosed cases. Therefore most patients die with this disease rather than from it (Nelson et al., 2008). The treatment of localized PC includes surgery or radiation therapy. In cases of relapse after a definitive treatment or in patients with locally advanced (T3b to T4) or metastatic disease (N1 or M1), the standard treatment includes the androgen-deprivation therapy (ADT; Huggins and Hodges, 2002). The ADT aims to reduce the circulating levels of testosterone around or below levels present in castration (<50 ng/ml). The reason behind the use of hormone therapy in the PC treatment is that the tumor growth is initially dependent on androgens. Androgens stimulate the tumor cells proliferation and inhibit their apoptosis. Conversely, ADT reverses the equilibrium in favor of the second one (**Figure 2**).

**FIGURE 3:**
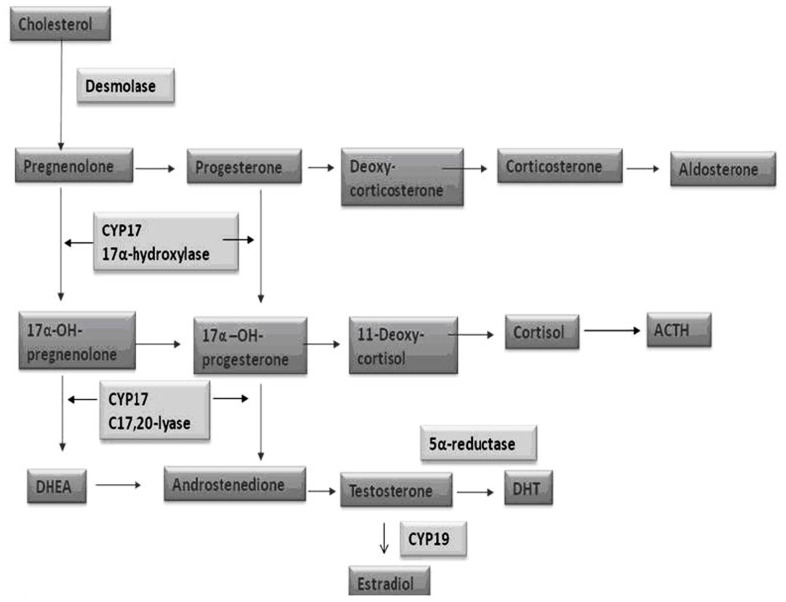
**Steroid biosynthesis**.

The androgen deprivation (AD) can be achieved by the use of LHRH analogs (medical castration) or bilateral orchiectomy (surgical castration). Through the circulating levels of testosterone and dihydrotestosterone (DHT) reduction, the ADT indirectly affects the androgen receptor (AR), which represents the real target of this therapy. The androgen biosynthesis consists in a multi-step process taking place in man’s gonads and adrenals and in which many enzymes are involved. Cytochrome P450c17α, with both 17α-hydroxylase and 17,20-lyase activity, has a key role among these proteins (**Figure 3**).

**FIGURE 4:**
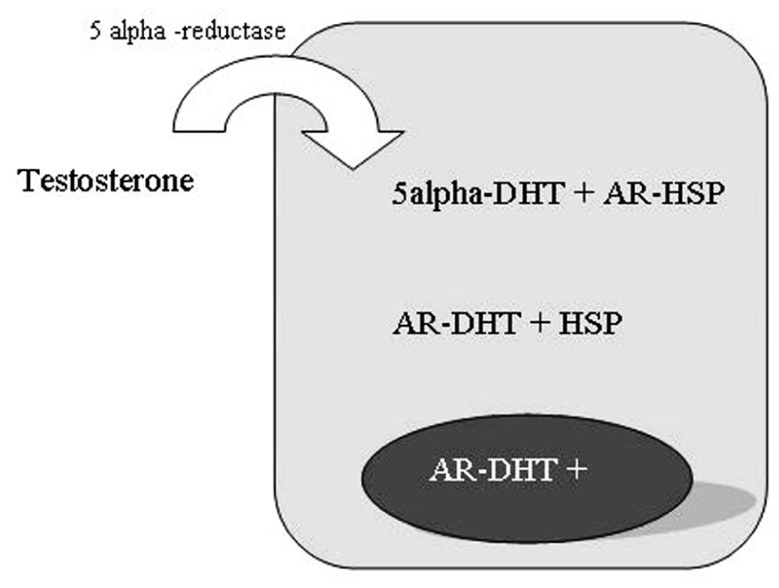
**5-alpha-reductase activity in prostate cells**.

The testosterone is the primary circulating androgen in man, chiefly produced by Leydig cells in the testes and converted to DHT in prostate cells by the enzyme 5-alpha-reductase (**Figure 4**; Steers, 2001).

The androgen DHT is more active having a fivefold higher affinity than testosterone for the AR. The androgen signal blockade kills PC cells through the induction of a programed cellular death. The effects of ADT are usually temporary and nearly all patients, initially sensitive to the androgen ablation therapy, have a disease progression after a 18–24 months medium term (Crawford, 1992). This probably results from the cancer cell clones selection, generated by the acquisition of critical somatic genome changes. Castration resistant PC (CRPC) is a lethal form of PC defined as a tumor which develops in spite of very low levels of androgens (below the threshold that defines castration). Preclinical investigation has led to advances in the understanding of the molecular basis of the CRPC. Various mechanisms have been proposed to explain how the tumor becomes able to maintain an incentive to grow in spite of very low serum levels of testosterone. This review aims to provide an overview about the genetic and epigenetic alterations having a crucial role in the carcinogenesis and in the disease progression, toward a more aggressive phenotype (Ruijter et al., 1999; Feldman and Feldman, 2001). In particular, we focused on the role of the AR, on its signaling cascade and the on its clinical implications that the knowledge of these aspects would have on the hormonal therapy, on its failure and toxicity.

## GENOMIC ALTERATIONS

Genomic alterations with a potential involvement in PC include somatic mutations, gene deletions or amplifications, chromosomal rearrangements. In the natural history of PC these alterations are probably accumulated over a period of several decades. The first studies on the molecular changes, with a potential crucial role in the development of PC, have identified chromosomal abnormalities frequently reported in PC patients, such as the gain of genetic material in 7p, 7q, 8q, and Xq and losses in 8p (Helcomb et al., 2009), 10q, 13q, and 16q. Some studies have shown that a chromosomal translocation involving TMPRSS2 (PSA-regulated gene transmembrane protease, serine 2), an androgen-responsive gene and a member of the ETS family of transcription factors (ERG, ETV1, ETV4, or ETV5) is present in over 60% of localized and metastatic prostate tumors and in 20% of PIN (Tomlins et al., 2005). ETS proteins cooperate with other transcription factors in the regulation of various cellular functions such as proliferation, differentiation, angiogenesis, apoptosis, and carcinogenesis. The ERG gene is the most common fusion partner with TMPRSS2. The incidence of the TMPRSS2–ERG fusion is almost 60% in PC. Both genes are localized on chromosome 21q22 and the fusion takes place by an interstitial deletion. Translocations involving other ETS family members occur rarely. Seventeen different TMPRSS2–ERG fusions, involving different regions of the TMPRSS and the ERG, have been identified. Eight of these fusions do not encode functioning ERG proteins for the introduction of a premature stop codon. Nine give rise to functional fusion products: two encode the normal ERG protein, six truncated ERG proteins, and one is a new protein resulting from the TMPRSS2 and the ERG fusion. The biological consequence of these different products is unknown. The TMPRSS–ERG translocation is considered an early event in the development of PC; it is not present in prostate benign lesions or hyperplasia. However, this is probably not the initial event as it is observed only in 20% of the PIN and it does not seem to have a direct role in the progression to adenocarcinoma. These data suggest that additional genetic mutations must occur. Aberrations were also identified in signal pathways of growth factors (NKX3.1, PTEN, c-MYC, and p27) and in the AR axis as determinants of the cancer cell phenotype. The LOH of 8p21.2 is found in 60% of the PIN and 85% of PC. The transcription factor NKX3.1 AR-regulated, whose normal function is to facilitate the terminal differentiation of prostatic epithelial cells, maps in this location. It has been suggested that the NKX3.1 is a gatekeeper tumor suppressor gene, similar to the APC in the colon cancer, to the VHL in renal clear cell carcinoma and to the RB in retinoblastoma. In contrast to peculiar tumor suppressor genes, however, allele of the NKX3.1 has not changed and the loss of heterozygosity determines a reduction of protein levels. The amplification of chromosome 8q24 associated to the over-expression of the transcription factor MYC is more frequently observed in PIN and in localized or metastatic PC. The decrease of p27, a cell cycle inhibitor, is correlated with an increase in histological grade of PC and with the risk of relapse. The LOH 12q12-13 containing p27 is present in 20% of localized disease cases and in approximately 50% of metastatic disease (Mackinnon et al., 2009). Also, single-nucleotide polymorphisms (SNPs) in key genes have been hypothesized to be associated with PC risk, outcome, and responsiveness to therapies. With respect to this topic, a meta-analysis based on 38 studies involving 34,782 cases of PC and 38,626 controls, suggested that the SNP of the CYP17 gene, rs743572, may be significantly associated with the risk of PC in the Black population (but not in Caucasian and Asian; Wang et al., 2011). Wright et al. (2010) affirmed that men with genetic variants in the CYP17 may differ in responsiveness to drugs from the wild-type gene carriers. This may have critical effects in regulating response of PC to the new CYP17 inhibitors like the Abiraterone acetate.

## EPIGENOMIC ALTERATIONS

Epigenetic changes are all the heritable changes in gene expression not resulting by alterations in the DNA sequence (Berger et al., 2009). The well-known epigenetic mechanisms are DNA methylation, changes to chromatin and alterations of microRNAs expression. Considerable evidences exist that a global DNA hypomethylation occurs late in PC contributing to the disease progression by promoting genomic instability (Chin et al., 2011). The inactivation of specific genes, caused by silencing their transcription through epigenetic alterations, is largely known as one of the mechanisms which can contribute to carcinogenesis. Among these epigenetic alterations in PC, the aberrant methylation of DNA in the promoter region of some genes (like GSTP – pi-class glutathione *S*-transferase) is a significant event (Lee et al., 1994). The somatic inactivation of the GSTP, which is a gene involved in detoxification, is the result of an aberrant methylation (hypermethylation) of CpG islands located in the gene promoter. As a result of this alteration the cells are more prone to accumulate additional mutations. This event seems to be involved in an early stage of the prostatic carcinogenesis: it is present in 70% of high-grade PIN and in more than 90% of adenocarcinomas (Nakayama et al., 2003; Kang et al., 2004). Li et al. (2001) observed that the degree of hypermethylation of E-cadherin gene (a gene in maintenance of normal cell architecture involved) in PC tissue was correlated with the pathological stage. They found an hypermethylation of E-cadherin gene promoter into the 30% of low-grade and the 70% of high-grade tumor tissues, respectively. Furthermore, hypermethylation of the p16 gene promoter results in a proliferative advantage of the cell clone so modified, with obvious implications, both in carcinogenesis and in the disease progression (Jarrard et al., 1997; Gu et al., 1998). In about 66% of PC the expression of the PTEN, an inhibitor of the PI3K/Akt pathway, is reduced or absent, with consequent activation of the PTEN/AKT/mTOR pathway. PTEN loss of function can result from deletion, mutation or epigenetic gene silencing. The PTEN epigenetic transcription silencing is often consequence of the CpG islands methylation located in its promoter region and would have a role in disease progression (Cairns et al., 1997; Suzuki et al., 1998). In some PC, the DNA hypermethylation could be also involved in the regulation of the AR expression. Suzuki et al. (2003) data showed that in 30% of hormone-refractory PC, the expression of the AR may be lost as a result of the AR promoter gene hypermethylation. Similarly the hypomethylation and consequent upregulation of genes like heparanase, urokinase may contribute to tumor cell invasion and metastasis (Hulett et al., 1999; Pakneshan et al., 2003). More examples of genes frequently silenced in PC are the APC, the MGMT, and the MDR1 (Kang et al., 2004).

## CASTRATION RESISTANT PROSTATE CANCER AND AR

The issue of *androgen independence* has been recently reviewed in light of the new knowledge achieved, so much so that today the term of *resistance to castration *is preferred. It is known that in the process of PC progression, the AR signaling axis maintains a decisive role: PC cells, once become resistant to castration, can evade the ADT cell growth inhibition and continue to express androgens-regulated genes even with castration serum levels of androgens. This should be partly explained by genetic alterations on the AR. As demonstrated in literature, AR alterations are observed in more than 50% of metastatic PC cases and tend to be rare in limited disease (Marcelli et al., 2000). It is yet unclear when the tumor acquires genetic alterations leading to castration resistance. While some studies suggest that tumor acquires these genetic alterations regardless of the ADT, other data highlight that the ADT would exert selective pressure on tumor cells. In this way the ADT would select cell clones able to grow independently from androgens, thanks to AR mutations (Taplin et al., 1995). The AR is a nuclear transcription factor whose gene belongs to the steroid–thyroid–retinoid nuclear receptor super-family. The AR gene is located on the X chromosome and contains eight exons. It encodes a protein of 919 amino acids. The AR is composed of four different domains: the N-terminal domain (NTD), the deoxyribonucleic acid-binding domain (DBD), the hinge region, and the ligand-binding domain (LBD; Lonergan and Tindall, 2011). AR aberrations correlated to the long-term failure of the ADT include the AR amplification/over-expression, the alternative source of androgens, the mutated AR or promiscuous AR, the over-expression of AR co-regulators, the AR activation by CK or growth factors. Nevertheless, the AR continues to be expressed even in disease advanced stages, regardless sensitivity to hormonal therapy (van der Kast et al., 1991; Hobisch et al., 1995).

### AR AMPLIFICATION/OVER-EXPRESSION

The amplification of the AR gene has been associated with endocrine therapy failure. In a Finnish study on 54 patients, this specific alteration was found in about 30% of locally recurrent or metastatic hormone-refractory tumors, while absent in the untreated ones. Koivisto et al. (1997) concluded that AR amplifications occurs exclusively during the disease progression after the ADT and it is more frequent in tumors with an initial good response to the hormone therapy. Indeed, tumor cells in this case would be more critically dependent on androgen than in patients primarily resistant to ADT. Also, recurrent tumors with the AR gene amplification treated with a first-line hormonal monotherapy, could benefit from a second-line combined androgen blockade, more than patients without this AR alteration, although this does not mean a gain in survival (Palmberg et al., 2000). Gregory et al. (2004) also confirms that the basis of castration resistance may consist in an hypersensitivity of the AR, resulting from its over-expression, increased stability and intranuclear localization.

### ALTERNATIVE SOURCE OF ANDROGENS

In spite of extremely low levels of circulating androgens, the CRPC progression remains dependent on the androgen-driven activity and PC cells keep the AR pathway active in different ways. An hypothesis standing to explain this observation is that alternative sources of androgenic steroids exist. Pioneer works, confirmed by recent researches in this area, indicated that intraprostatic amount of testosterone and DHT remains moderately high despite their castration serum levels (Liu et al., 1985; Labrie, 2004; Titus et al., 2005). Locke et al. (2008) hypothesized that androgens *de novo* synthesized within prostatic tumor tissue may drive the CRPC progression in the absence of testicular androgens. Also they argue that some enzymes necessary for androgens synthesis (SRD5A1, RDH5, ARK1C1,2,3) are up-regulated in PC cells during the CRPC progression. Montgomery et al. (2008) remarked this theory showing that all the enzymes involved in the biosynthesis of testosterone and DHT were expressed in the majority of metastatic CRPC examined in his study. This would explain how the decrease of DHT in prostate tissue after the ADT may not be proportional to decreased levels of circulating testosterone (60 vs. 95% respectively; Labrie et al., 1986). What also appears to be assumed is that the adrenal steroids peripheral conversion may be sufficient to the maintain androgen signal, resulting in the tumor growth and in the ADT failure. This is the rationale behind the use of the combination therapy with antiandrogen plus LHRH analog and the basis for use of CYP17 inhibitors like ketoconazole (use limited by its toxicity; Trachtenberg and Pont, 1984), recently the abiraterone acetate (a potent inhibitor of the CYP17α-hydroxylase; De Bono et al., 2011) and Orteronel (TAK-700; Yamaoka et al., 2012).

### MUTATED OR PROMISCUOUS AR

Androgen receptor genetic changes leading to aberrant functioning of the AR pathway may underlie the development of resistance to castration, allowing tumor cells to avoid the normal stimuli to growth. Largely, we are talking about somatic mutations in the AR gene, resulting in an increase of the potential ligands which bind and activate the AR receptor. The AR somatic mutation would include missense mutations like T877A and L701H. The T877A missense mutation has been described for the first time in LNCaP cell lines and affects the LBD of the AR. This alteration allows to ligands different from testosterone and DHT, such as progestins, estrogens, and antiandrogens to activate the AR. This may provide a molecular basis to “withdrawal syndrome” (i.e., patients treated with maximal androgen blockade, can benefit from the stop of antiandrogen (Kelly and Scher, 1993; Small and Srinivas, 1995). Zhao et al. (2000) described how the L701H mutation in conjunction with the T877A mutation of the LBD of the AR, could involve stimulating mutant cells growth by glucocorticoids (cortisone, cortisol). This may have important implications in clinical practice when you choose the glucocorticoid to be used in therapy.

### OVER-EXPRESSION OF AR CO-REGULATORS

The AR activates its target gene transcription in response to the androgen stimulus. Its transactivation activities are modulated by co-regulators. Some evidences support the importance of co-regulators in the carcinogenesis and the development of hormone-resistance in PC. AR co-regulators consist of almost 200 proteins able to enhance (co-activators) or repress (co-repressors) the transcriptional activity of the AR. Genes mutation or the aberrant expression of this molecules may participate in PC progression (Gao et al., 2002). Several findings suggest that the NCOA2 gene (a nuclear receptor co-activator) would play the role of the potential oncogene in PC. Taylor et al. (2010) hypothesized that its amplification or mutation in primary and metastatic PC enhances the androgen-dependent AR transcriptional activity. The steroid receptor co-activator (SRC) family includes the SRC-1, the SRC-2, and the SRC-3. Literature shows that the SRC-1 is over-expressed in ADT-refractory PCs. Gregory et al. (2004) found that the SRC-2 is also over-expressed in recurrent PCs. This change may contribute to PC relapse after endocrine therapy. There is no clear evidence about the possible roles of the SRC-3 in prostate tumor development and progression. The steroid receptor RNA activator (SRA) is another co-activator for steroid receptors which functions as a RNA transcript in a ribonucleoprotein complex containing the SRC-1. Kawashima et al. (2002) showed that the SRA expression is higher in androgen-independent PC cell lines (PC-3) compared to the androgen-dependent ones (DU-145, LNCaP). The AR-associated protein ARA70 can interact with the AR and modulate its transcriptional activity in response to the androgens stimulation. It must also be reported that the ARA70 could facilitate the agonist activity of antiandrogens like bicalutamide (Miyamoto et al., 1998). In normal prostatic epithelium the ARA70 is highly expressed, while studies showed that the ARA70 expression was decreased or suppressed by methylation in androgen-dependent PC cell lines (DU145). Its expression seems to be regulated by both ER and AR in PC cells (Tekur et al., 2001).

### THE AR ACTIVATION BY CK AND GROWTH FACTORS

The possibility of a cross-talk between the AR pathway and intracellular signaling cascades activated by IGF-1, KGF, and EGF, leading to transcription of androgen controlled genes, in the absence of the ligand exists (Culig et al., 1994). The ErbB or HER receptor network is frequently alterated in solid tumors. The HER kinase family includes: the epidermal growth factor receptor (EGFR or ErbB1), the human epidermal growth factor receptor 2 (HER2), the ErbB3 (HER3), and the ErbB4 (HER4). The HER2 is the only one in a fixed open conformation, other members need to bind to the ligand to form active dimer instead (Solit and Rosen, 2007). In breast cancers, the ErbB2 over-expression correlates with estrogen independence. In the ErbB2 over-expressed breast cancers therapy the anti-HER2 monoclonal antibody, trastuzumab (Herceptin) can be used. Similar to the breast cancer case, or even in PC (Signoretti et al., 2000), it may exist a connection between the over-expression of the HER2/neu (also known as ErbB2) and the progression to castrate resistant disease. The activation of the HER2 signaling cascade may lead to constitutive activation of the AR (Craft et al., 1999; Yeh et al., 1999). Studies suggest that the ErbB receptor activation may be important in the growth and in the survival of both androgen-dependent and androgen-independent PC (Agus et al., 2002). In particular, the ErbB2–ErbB3 signaling has been implicated in enhancing the AR signaling through modulation of its transcriptional activity and its degradation in the presence of low androgen levels (Mellinghoff et al., 2004). Wen et al. (2000) showed in LNCaP cells that the HER2/neu activates Akt (protein kinase B) and in this way promotes the PC cells survival and growth in absence of androgens. These conditions may open new ways to PC therapy. However, when trastuzumab (Lara et al., 2004; Ziada et al., 2004) and pertuzumab (a second generation of anti-HER2 monoclonal antibody; De Bono et al., 2007), as well as the EGFR tyrosine kinase inhibitors (TKIs) gefitinib (Canil et al., 2005) and erlotinib (Gravis et al., 2006) have been tested in PC, it was observed a non-significant single-agent activity. These results suggest that these targets may be of secondary importance or of primary importance only in few cases of PC. According to the disappointing results obtained by the single use of these molecules, some researchers attempted to test them in combination. These therapeutic strategies aim to get the return of prostatic cancer cells to the androgen sensibility. Distinct phase II studies, show that three EGFR inhibitors have been combined with docetaxel. In single-arm studies of the gefitinib or erlotinib, PSA and tumor responses were modest, although the erlotinib showed favorable survival (24.6 months) with increased toxicity (Gross et al., 2007; Salzberg et al., 2007). Recent evidences also suggest that the HER-2 expression confers an increased risk of CNS metastases in the metastatic CRPC (Gernone et al., 2011). Other factors not directly related to the AR are certainly involved in the carcinogenesis and progression of PC. Data from some studies point to the potential role of proteins involved in the regulation of vascular permeability and endothelial proliferation. In 2006, a study shown that the endocrine gland-derived vascular endothelial growth factor/prokineticin 1 and 2 (EG-VEGF/PK1 and 2) and their receptors were expressed after the transition from benign to malignant prostatic glandular epithelium and that their levels increased with increasing histological grade (Pasquali et al., 2006).

## TREATMENT FOR CASTRATION-RESISTANT PROSTATE CANCER

Currently, chemotherapy represents one of the possible treatment options in the setting of the CRPC, providing modest survival and palliative benefits. However, many other therapeutic options are now available. With the FDA approval of Docetaxel in 2004 prospects on the therapeutic possibilities for this type of cancer has gradually expanded. Docetaxel-based chemotherapy (given every 3 weeks at a dose of 75 mg/m^2^ along with a corticosteroid) as standard treatment in the CRPC is based on the survival advantage (approximately 3 months in median OS when compared with mitoxantrone and prednisone) observed in two independent phase III studies (TAX 327 trial and SWOG 99-16 trial). However, most men treated with this drug experienced progression of their disease within 1 year from the start of chemotherapy. Between 2010 and 2011 the cabazitaxel, the sipuleucel-T and the abiraterone acetate were also approved by the FDA for treatment of metastatic CRPC. In addition, a novel new bone-targeting monoclonal antibody, denosumab (Xgeva), and an LHRH antagonist, degarelix (Firmagon), have been introduced into clinical practice. Cabazitaxel is a microtubule inhibitor approved by FDA for the treatment of patients with CRPC progressed after docetaxel. Cabazitaxel, in a phase III multicenter study, in which it was compared with mitoxantrone, showed a statistically significant advantage in terms of median overall survival (15.1 vs. 12.7) and PFS (2.8 vs. 1.4; De Bono et al., 2010). Also Abiraterone, in a phase III study, has been shown to improve the outcome of men progressing during or after a docetaxel-based chemotherapy treatment in which it prolonged the patients median survival to 4 months if compared to placebo (De Bono et al., 2011). Abiraterone (a potent and irreversible inhibitor of the CYP17A) is designed to treat the CRPC by inhibiting the production of androgen in the testis, in the adrenal glands, and in prostate tumor itself. Since there are no head-to-head comparisons to guide us in choosing between these two drugs, largely, patients for these therapies are selected according to the different side effect profile of these agents. One trial is currently ongoing looking at the comparison of abiraterone plus prednisone vs. placebo and prednisone in asymptomatic or mildly symptomatic metastatic CRPC who have not received chemotherapy. The Sipuleucel-T is an autologous cellular immunological agent, obtained by leukapheresis and cultured (activated) with a recombinant human protein (PAP-GM-CSF) consisting of prostatic acid phosphatase linked to the granulocyte-macrophage colony-stimulating factor, that shown to improve survival in minimally symptomatic patients. The Sipuleucel-T is thought to work through APCs to stimulate the T cell immune response targeted against the PAP, an antigen that is highly expressed in most PC cells. The mechanism for immunotherapy of PC, in general, is that cancer cells contain antigens of the prostate that can be recognized by the immune system (through the APC and T cells) allowing a selective killing of PC cells. T cells respond to small peptides derived from intracellular proteins and are present on specialized molecules on the surface of cancer cells. PC cells can be recognized by virtue of containing new proteins, formed as a result of mutations in somatic gene translocations, or to express proteins lineage, representing differentiation prostate cells (Kantoff et al., 2010a). Today new agents are being evaluated for men with metastatic CRPC in both first-line (in combination with docetaxel) and second-line treatment (in men progressing during or after treatment with docetaxel). Phase III clinical studies are ongoing on molecules with different targets: the androgen signaling pathway (MDV3100, Scher et al., 2010; TAK-700, Dreicer et al., 2010) has demonstrated significant activity in phase I and II studies), immunoregulatory pathways (Ipilimumab, the Prostvac-VF-Tricom (Kantoff et al., 2010b), the Src (dasatinib; Araujo et al., 2009), the Met (cabozantinib), and the angiogenesis (aflibercept; Isambert et al., 2008, tasquinimod). Recently, attention has been paid to the possibility of radioimmunotherapy (RIT) use in PC. The prostate-specific membrane antigen (PSMA) is an integral cell-surface membrane protein and an ideal target for monoclonal antibody therapy. The anti-PSMA monoclonal antibody – radionuclide labeled (J591) has been studied in phase I and II studies. The most common radionuclides used have been 90-Yttrium and 177-Lutetium. This trials seem to suggest that the radiolabeled J591 has antitumor activity and is well tolerated. Phase I trial on chemotherapy and RIT combination is also ongoing (Milowsky et al., 2004; Bander et al., 2005; Tagawa et al., 2008,^2010^). However, resistance to the first-line chemotherapy occurs, inevitably, even in patients who initially responded. Studies were designed in order to overcome this obstacle in different ways. Some researchers attempted associating docetaxel to a molecule able to interfere with one or more mechanisms of chemotherapy resistance. The aim is to improve the efficiency of a well-known cytotoxic agent to carry out its task. On the basis of encouraging results of a phase II trial, Chi et al. (2009) employed this strategy in a phase III study using the antisense oligonucleotide: the custirsen (OGX-011). Evidences in literature also indicate that tumor microenvironment may be critically implicate in PC therapy resistance. In particular angiogenic growth factors like vascular endothelial growth factor (VEGF) may have a crucial role in mechanisms guiding bone metastatization and disease progression. So recently attention has been paid to the use of docetaxel in conjunction with stroma-targeting molecules.

## CONCLUSION

Nowadays treatment options available for the CRPC are many and new questions emerge. First, how these new drugs have to be administered in clinical practice. In the era of the personalized cancer therapies, the deep knowledge of underlying mechanisms of the castration resistance of PC has become a topic of primary importance. Research in this field aims to obtain the tools in order to choose the most effective and less toxic therapy for each patient. This is more evident if we think about the impact of the hormone therapy on patients with PC quality life. In this way risks and benefits, ratio of a single therapy, have a greater significance. New biomarkers able to predict which patients may really benefit from a specific therapy without being exposed to an unnecessary toxicity, are required. For the future it should be possible to understand the genetic background of our patients with regard to some critical enzymes which could influence both response and resistance to drugs, before starting a specific therapy. This chance could make it really feasible to personalize the therapy in individual patients.

## Conflict of Interest Statement

The authors declare that the research was conducted in the absence of any commercial or financial relationships that could be construed as a potential conflict of interest.
